# Nuclear Softness Promotes the Metastatic Potential of Large-Nucleated Colorectal Cancer Cells via the ErbB4-Akt1-Lamin A/C Signaling Pathway

**DOI:** 10.7150/ijbs.89481

**Published:** 2024-04-29

**Authors:** Yangkun Li, Qilin Li, Lei Mu, Yibing Hu, Chang Yan, Hui Zhao, Yulong Mi, Xiaolan Li, Deding Tao, Jichao Qin

**Affiliations:** 1 Molecular Medicine Center, Tongji Hospital, Tongji Medical College, Huazhong University of Science and Technology, Wuhan, 430030, China.; 2 Department of Surgery, Tongji Hospital, Tongji Medical College, Huazhong University of Science and Technology, Wuhan, 430030, China.; 3 Department of Breast Surgery, Peking University Shenzhen Hospital, Shenzhen, 518000, China.; 4 Department of Gastrointestinal Surgery, Peking University Shenzhen Hospital, Shenzhen, 518000, China.; 5 Department of Surgical Oncology, Fujian Provincial Hospital, Shengli Clinical Medical College of Fujian Medical University, Fuzhou, 350013, China.; 6 Department of Gastrointestinal Surgery, the First Affiliated Hospital, Zhejiang University School of Medicine, Hangzhou, 310003, China.

**Keywords:** Colorectal cancer, Tumor metastasis, Nuclear size, Nuclear stiffness, Lamin A/C

## Abstract

Abnormal nuclear enlargement is a diagnostic and physical hallmark of malignant tumors. Large nuclei are positively associated with an increased risk of developing metastasis; however, a large nucleus is inevitably more resistant to cell migration due to its size. The present study demonstrated that the nuclear size of primary colorectal cancer (CRC) cells at an advanced stage was larger than cells at an early stage. In addition, the nuclei of CRC liver metastases were larger than those of the corresponding primary CRC tissues. CRC cells were sorted into large-nucleated cells (LNCs) and small-nucleated cells (SNCs). Purified LNCs exhibited greater constricted migratory and metastatic capacity than SNCs *in vitro* and *in vivo*. Mechanistically, ErbB4 was highly expressed in LNCs, which phosphorylated lamin A/C at serine 22 via the ErbB4-Akt1 signaling pathway. Furthermore, the level of phosphorylated lamin A/C was a negative determinant of nuclear stiffness. Taken together, CRC LNCs possessed greater constricted migratory and metastatic potential than SNCs due to ErbB4-Akt1-mediated lamin A/C phosphorylation and nuclear softening. These results may provide a potential treatment strategy for tumor metastasis by targeting nuclear stiffness in patients with cancer, particularly CRC.

## Introduction

Colorectal cancer (CRC) is the third-leading cause of cancer mortality, and patients with CRC generally die from metastatic lesions 1. Tumor metastasis involves a multi-step process characterized as the metastatic cascade, comprising invasion, intravasation, circulation, extravasation, and colonization 2. During these processes, tumor cells migrate through confined pores in the extracellular matrix (ECM) that vary from 1 to 20 μm in diameter 3. The confined matrix provides mechanical cues to modulate the physical properties of the tumor cells, especially their stiffness, to squeeze through the tiny pores 4. Notably, the nucleus is the largest organelle, with a diameter of 5 to 20 μm, and the most difficult part to deform 5. During confined cell migration, tumor cell motility is halted at pore sizes smaller than 7 μm^2^ due to a lack of nuclear movement 6. Therefore, nuclear squeezing is the rate-limiting factor in the confined migration of tumor cells.

Nuclear squeezing is closely related to its physical properties, particularly nuclear size and stiffness. Nuclear size is heterogeneous in various cancers 7-9. Cancer cells with enlarged nuclei indicate a more aggressive metastatic disease 10. Preoperative treatment of patients with breast cancer with anti-estrogen therapy results in decreased nuclear size and metastasis 11. A large nuclear region is also a predictor of CRC recurrence and metastasis 12. Along with nuclear size, nucleus stiffness is also associated with cancer metastasis. Softening of the nucleus enhances the invasion of human breast cancer cells 13. Increased nuclear stiffness inhibits the fast amoeboid migration of melanoma cells 14. However, the association between nuclear size and stiffness and the mechanisms by which large-nucleated cells or soft-nucleated cells facilitate metastasis are unclear.

Nuclear physical properties are regulated by the nuclear structure, which comprises the nuclear envelope (NE), lamina surrounding the nucleoplasm, the genome, and subnuclear bodies 15. The nuclear lamina is a dense meshwork of intermediate filament proteins that confers mechanical support to the nucleus 16. Lamin A/C, the major component of the lamina, modulates the response of cells to spatial confinement by conferring viscoelastic properties to the nucleus and may indicate cell-specific adaptations to the mechanical demands of the local microenvironment 17-19. Elevating lamin A/C inhibits the migration of melanoma cells, while loss of lamin A/C results in abnormal nuclear shape and increased confined migration 14, 18. Lamin A/C is also reported to be phosphorylated and provides the molecular basis for nuclear softening and deformation 20, 21.

Erb-B2 receptor tyrosine kinase 4 (ErbB4; also referred to as HER4) is a member of the ERBB family, which is involved in cell cycle, proliferation, and apoptosis 22, 23. In addition, ErbB4 activates the Akt cascade and focal adhesion kinase, leading to the tumor spread of Ewing sarcoma 24. Notably, ErbB4 is elevated or hyperphosphorylated in CRC and associated with aggressive CRC diseases 22, 25. Once activated, the soluble intracellular domain of ErbB4 translocates to the nucleus and modulates nuclear signaling 26. The ERBB family also converts mechanical stimuli in the microenvironment into biochemical signals that promote tumor progression 27. However, it is not clear whether ErbB4 can directly alter the mechanotransduction and nuclear structures.

In the present study, CRC cells were sorted into large-nucleated cells (LNCs) and small-nucleated cells (SNCs), and the association between the confined migration and nuclear stiffness of LNCs and SNCs was investigated. Of note, the underlying mechanism by which ErbB4 regulates nuclear stiffness was explored.

## Materials and Methods

### Cell culture

The LoVo, SW620, HCT116, SW48, and SW480 human colon cancer cell lines were purchased from the cell bank of the Chinese Academy of Sciences (Shanghai, China). The XhCRC cells were established as previously described 28. Xenograft tumors were minced and incubated in DMEM/F12 (Thermo Fisher Scientific; Waltham, MA, USA) containing 1.5 mg/mL collagenase IV (Thermo Fisher Scientific), 20 µg/mL hyaluronidase (Sigma-Aldrich; Merck KGaA, Darmstadt, Germany), and 1% penicillin/streptomycin (Thermo Fisher Scientific) at 37 °C for 1 h. To remove red blood cells, the cells were treated with red blood cell lysis buffer (Thermo Fisher Scientific) on ice for 10 min, then washed twice with PBS. Isolated single cells were stained with EpCAM and subjected to fluorescence-activated cell sorting (FACSAria II, BD Biosciences; San Jose, CA, USA) to purify EpCAM-positive tumor cells. All cell lines were cultured in DMEM (Thermo Fisher Scientific) with 10% FBS (Thermo Fisher Scientific) and incubated at 37 °C with 5% CO_2_ in a cell culture incubator. Mycoplasma was routinely tested using a mycoplasma detection kit (Thermo Fisher Scientific).

### Pharmacological inhibitors

In selected experiments, cells were treated with the following pharmacological agents and corresponding vehicle controls. The following reagents were purchased from MedChemExpress (NJ, USA): trichostatin A (TSA) (HY15144; 2 μM, 24 h), methylstat (HY15221; 2 μM, 48 h), Akt kinase inhibitor (Akti) (HY10249A; 0.5 μM, 0.5 h), and paclitaxel (Taxol) (HYB0015; 20 nM, 12 h).

### Immunohistochemistry

Immunohistochemistry was performed as previously described 29. Carcinoma specimens were gathered from patients with CRC. Mouse livers were gathered from the *in vivo* assays. Four-millimeter-thick section slides were stained with hematoxylin and eosin (HE). Five fields were selected from each slide by two experienced pathologists. All human CRC tissue studies were performed under the protocols approved by the Ethical Committee of Tongji Hospital, Tongji Medical College, Huazhong University of Science and Technology (HUST) (IRB ID 20141106). The clinical history of the human subjects was listed in Table S1.

### Fluorescence-activated cell sorting (FACS)

Cells were previously infected with the LentiGuide-puro-nuclear localization sequence (NLS)-green fluorescent protein (GFP) lentiviral vector, a gift from Daniel Durocher (Addgene; Watertown, MA, USA) 30. FACS was performed according to the manufacturer's instructions using FACS Aria II Cell Sorter (BD Biosciences), followed by flow cytometric analysis using Diva software (BD Biosciences). All cells were first passed through a 30-μm cell strainer to remove adherent cells. Generally, debris was removed by gating in the light scatter versus forward scatter (FSC) plots. The top 5% and bottom 5% of gated cells were sorted based on fluorescence signal width.

### Cell cycle analysis

Cells (1 × 10^6^/mL) were gently vortexed with ice-cold 75% ethanol and then fixed overnight at 4 °C. Subsequently, the cells were rinsed and resuspended in 200 µL of cold PBS. Next, the cell suspension was incubated with 20 µL RNase A (Thermo Fisher Scientific) for 30 min at 37 °C and then with 200 µL propidium iodide (PI) (Thermo Fisher Scientific) for 20 min at 4 °C. Analyses were conducted with FACSVerse (BD Biosciences).

### Immunofluorescence (IF)

Cells were seeded on glass-bottomed Petri dishes overnight and subsequently fixed with 4% paraformaldehyde for 10 min at room temperature. The fixed cells were permeabilized with 0.1% Triton X-100 for 10 min, blocked with 1% bovine serum albumin (BSA) for 1 h, and incubated with primary antibodies at 4 °C overnight. The cells were then incubated with secondary antibodies for 1 h and DAPI for 10 min at room temperature. Visualization was done using a fluorescence microscope (Olympus BX53 or CKX41) or a confocal laser scanning microscope (Olympus FV1000). Antibodies were listed in Table S2.

### Cell migration assays

Transwell permeable supports with 3.0, 5.0, and 8.0 μm pore polycarbonate membranes were purchased from Corning (NY, USA). Cells (5 × 10^4^/200 µL) were resuspended in serum-free medium and seeded into the top chamber. 650 μL of medium containing 10% FBS were added to the bottom chamber. After being incubated for 12 h, cells on the top side of the upper chamber were removed by swabbing. Cells attached to the back side of the upper chamber were stained with a 0.1% crystal violet solution and observed by a light microscope (Olympus BX53). Three visual fields were randomly chosen to calculate the number of migrated cells.

### *In vivo* assays

Animal experiments were performed under protocols approved by the Institutional Animal Care and Use Committee of Huazhong University of Science and Technology (IACUC No. S2348). Female NOD/SCID and nude mice (4 weeks old) were purchased from Beijing HFK Bioscience. Mice were randomly divided into 3-5 mice per group and weighed to confirm that the weight difference was no more than 2 g. For the liver metastasis assays, 1 × 10^6^ cells were resuspended in 100 μL PBS and injected into the spleen of female nude mice. According to IACUC policy, the body condition score (BCS) was assessed twice a week, and mice with a BCS of <2/5 were euthanized. After 8-10 weeks, all surviving mice were euthanized by CO_2_ inhalation (30% vol/min). Mouse liver was harvested, and liver metastatic lesions were counted and confirmed by HE staining.

### Atomic force microscopy (AFM)

Samples were probed with Asylum-MFP3D-Bio-AFM mounted on an Olympus IX-71 inverted optical microscope platform. Stiff pyramidal cantilevers with a nominal spring constant of ~10 pN/nm tips (32 kHz; TR400PB; Asylum Research) were used to measure nuclear stiffness. Indentation data of 500-2,000 nm were fitted to nuclear stiffness measurements using the Hertz model to obtain estimates of nuclear stiffness 5, 31.

### RNA-sequencing (RNA-seq) analysis

RNA was extracted from CRC cells using Trizol (TaKaRa; Shiga, Japan) according to the manufacturer's protocol. RNA quality was determined using a 2100 Bioanalyzer (Agilent) and quantified using the Nanodrop 2000. Reverse transcription, library construction, and sequencing were performed using the Illumina Hiseq2000 platform at Majorbio Biotechnology (Shanghai, China). The library was prepared following Illumina-stranded mRNA preparation and ligation. Quality control and read mapping were conducted by the HISAT2 software. Differentially expressed genes were quantified using the transcripts per million (TPM) reads method. RSEM was used to quantify gene abundances. Differential expression analysis was performed using DESeq2. Genes with |log2 FC| ≥ 1 and *P* adjust < 0.05 were considered to be differentially expressed genes. Genes with TPM >1 were identified for Venn analysis. In addition, functional enrichment analysis was carried out by the online Majorbio I-Sanger Cloud Platform. Raw data was uploaded to the SRA database: PRJNA986600.

### RNA expression analysis

RNA was extracted from CRC cells using Trizol (TaKaRa), and cDNA was synthesized using PrimeScript RT Master Mix (TaKaRa) according to the manufacturer's protocol. Reverse transcription-quantitative PCR (RT-qPCR) was performed using SYBR Green PCR Master Mix (TaKaRa) and an ABI PRISM 7300 Sequence Detection System (Thermo Fisher Scientific). Expression data were uniformly normalized to the internal control *GAPDH*, and the relative expression levels were evaluated using the ΔΔCt method. Primers were listed in Table S3.

### Western blotting

Cells were lysed in NonidetP-40 (NP40) buffer with a complete protease and phosphatase inhibitor (Sigma-Aldrich). Protein concentration was determined using a BCA assay (Thermo Fisher Scientific). Protein samples (20 μg/per sample) were electrophoretically separated and transferred to 0.22 μm PVDF membranes (Millipore; Merck KGaA, Darmstadt, Germany). Molecular weight-specific membranes were blocked with 5% BSA in Tris-buffered saline for 1 h and incubated with specific primary antibodies at 4 °C overnight, followed by incubation with HRP-conjugated secondary antibodies for 2 h at room temperature. Finally, the membranes were visualized using ECL reagents (Thermo Fisher Scientific). Antibodies were listed in Table S2.

### Co-immunoprecipitation (CoIP) and quantitative IP (qIP)

Flag-Akt1 plasmid was purchased from MiaoLingBio (P42793; Wuhan, China). Wild-type and S-A mutant HA-lamin A/C plasmids were synthesized using Mut Express II Fast Mutagenesis Kit V2 (Vazyme; Nanjing, China) according to the manufacturer's protocol. Transfected cells (1 × 10^7^/mL) were lysed in an NP40 solution on ice for 30 min. After centrifugation at 4 °C, 13,000 × g for 10 min, the supernatant protein concentration was determined by a BCA assay (Thermo Fisher Scientific) and diluted to 1 μg/μL with NP40. 20 μL anti-HA magnetic beads or anti-Flag magnetic beads (HYK0201, HYK0207; MedChemExpress) were added to 700 μL protein supernatants. The mixture was incubated at 4 °C overnight. The beads were gathered and then boiled in 70 μL of SDS loading buffer for 10 min. A total of 10 μL per sample was loaded onto SDS-PAGE gels for Western blotting analysis and 60 μL per sample for mass spectrometry (MS) analysis.

### Mass spectrometry (MS) analysis

The protein sample was sent to the National Protein Science Facility, School of Life Science, Tsinghua University, China. Samples were digested, dried, and redissolved in 0.1% trifluoroacetic acid. Peptides were analyzed by an Orbitrap Fusion LUMOS Tribrid (Thermo Fisher Scientific). The MS data was searched against the target protein database from UniProt using an in-house proteome discoverer (Version PD 1.4; Thermo Fisher Scientific).

### Statistical analysis

Statistical analysis was performed using GraphPad Prism 8.0 software (La Jolla, CA, USA). The mean ± SD was used to present all values. Statistical tests were used as appropriate. Student's *t* test for two groups or ANOVA followed by a Tukey's test for multiple comparisons. *ERBB4* expression in datasets was examined using the *Kruskal-Wallis* test followed by a Dunn's test. The survival rate was analyzed using the Cox regression model. *P* < 0.05 was considered to indicate a statistically significant difference.

## Results

### Nuclear size is positively associated with the stage of CRC

**Figure 1:**
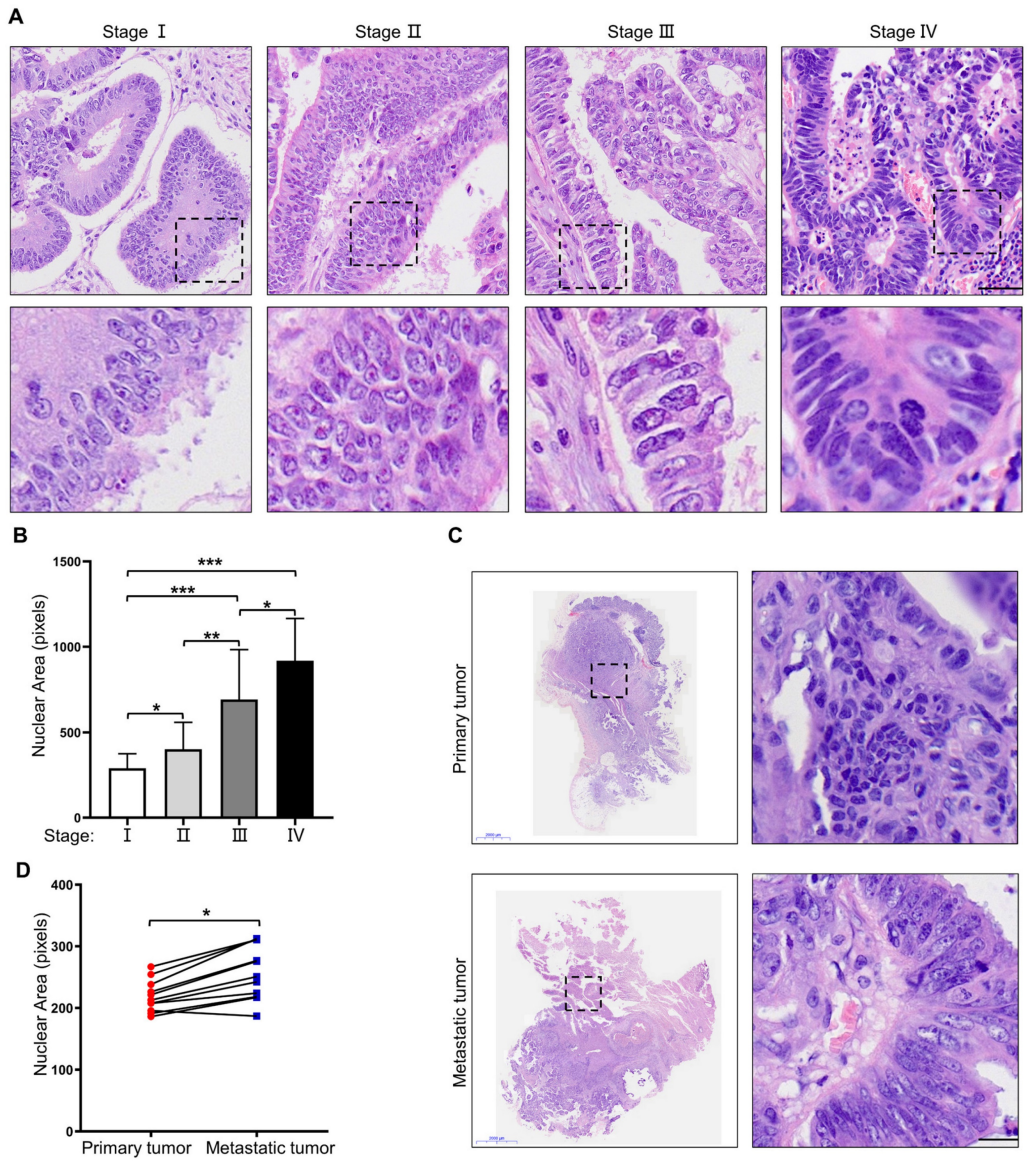
** Nuclear size is positively associated with the stage of CRC.** (A) HE staining of primary CRC specimens at different TNM stages. Scale bar: 20 μm. (B) Quantification of nuclear area in primary CRC specimens at different TNM stages. (C) HE staining of paired primary and metastatic CRC specimens. Scale bar: 2,000 μm and 20 μm. (D) Quantification of nuclear area in paired primary and metastatic CRC specimens. Results are presented as the mean ± SD. ^*^P < 0.05,^ **^P < 0.01, ^***^P < 0.001.

To investigate nuclear heterogeneity in colorectal tumors, HE staining was performed on primary colorectal tumors at different stages. The results showed larger nuclei in primary colorectal tumors at a more advanced stage (Figure 1A, B). Next, 11 pairs of primary colorectal cancer tissues and corresponding metastatic liver lesions were collected and analyzed for further proof. The results revealed larger nuclei in the metastatic liver lesions than those in primary tumor tissues (Figure 1C, D). Taken together, these results suggest that nuclear size is positively associated with the stage of CRC, and cells with large nuclei may possess increased metastatic potential.

### CRC cells with different-sized nuclei are capable of being prospectively sorted by FACS

**Figure 2:**
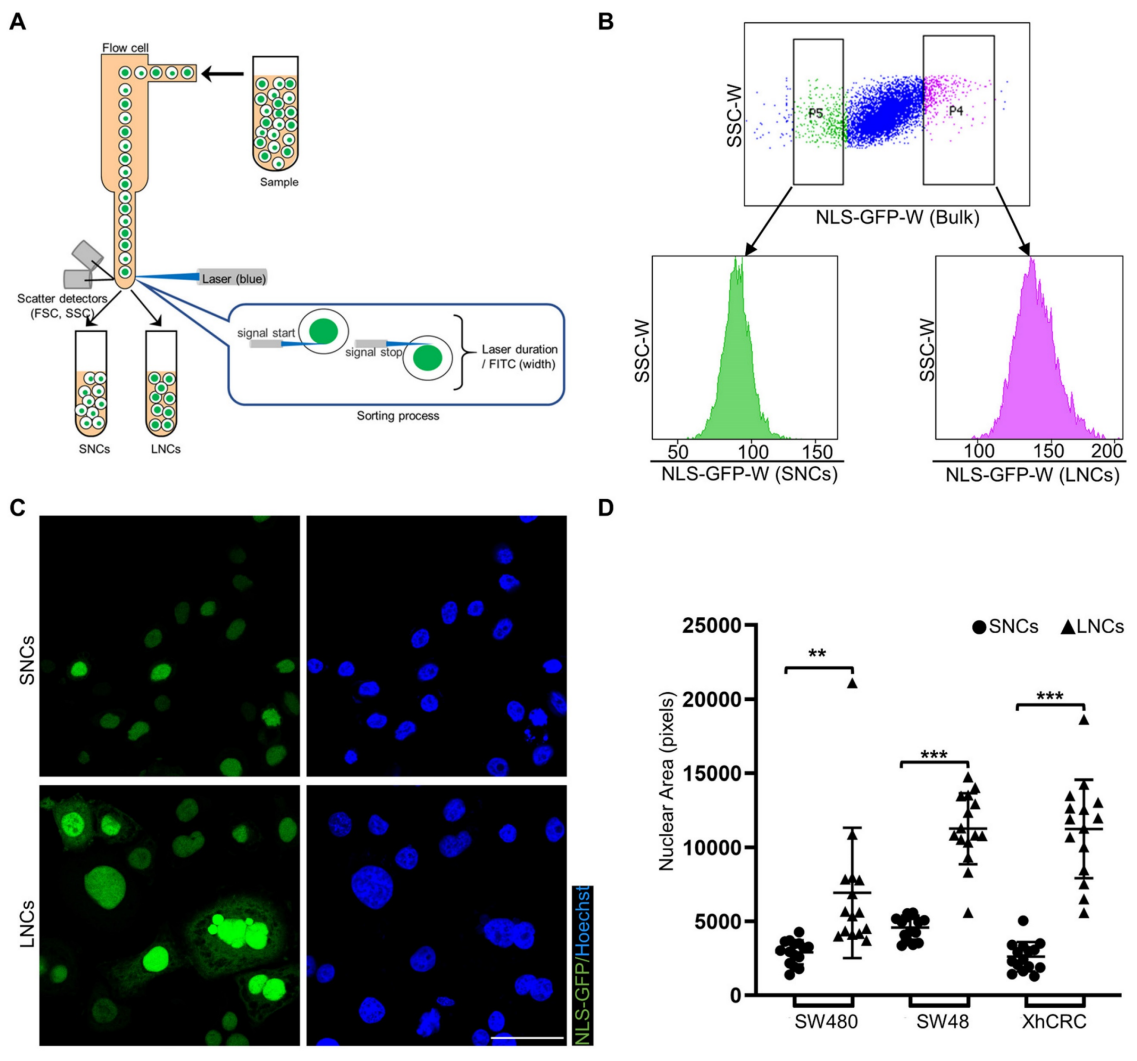
** CRC cells with different-sized nuclei are capable of being prospectively sorted by FACS.** (A) Schematic of FACS for CRC SNCs and LNCs. (B) Top-5% (LNCs) and bottom-5% (SNCs) NLS-GFP-Width SW480 cells were sorted out and performed for post-sorting analysis. (C) Immunofluorescence analysis of NLS-GFP and nuclei (Hoechst) in SW480 SNCs and LNCs. Scale bar: 20 μm. (D) Quantification of the nuclear area of CRC SNCs and LNCs. Results are presented as the mean ± SD. ^**^P < 0.01, ^***^P < 0.001.

NLS-GFP, a lentiviral vector that induces GFP expression and translocates GFP to the nucleus, is regarded as a marker of the nucleus 30, 32. NLS-GFP lentivirus was infected into several CRC cell lines, including SW48, SW480, SW620, HCT116, LoVo, and XhCRC cells (Figure S1A). Nuclear size was highly heterogeneous in SW480, SW48, and XhCRC cells compared with SW620, HCT116, and LoVo cells (Figure S1B, C). Therefore, SW480, SW48, and XhCRC cells were used in the subsequent experiments. To separate CRC cells into those with large vs. small nuclei (LNCs vs. SNCs), FACS was used to purify the top 5% (LNCs) and bottom 5% (SNCs) of cells based on the NLS-GFP width, which measured single-cell fluorescence duration (Figure 2A, B). Sorted LNCs showed significantly larger nuclei than SNCs (Figure 2C, D, and Figure S2A). For most tumor types, nuclear size change occurs without a corresponding change in cell size, and thus the nuclear-to-cytoplasmic volume (N/C) ratio is disrupted 33. Consistent with the previous study, flow cytometry analysis revealed no difference in cell size between LNCs and SNCs but a higher N/C ratio in LNCs (Figure S2B, C). Further cell cycle analysis indicated that there was no difference in the number of SNCs and LNCs in G0/G1, S, and G2/M phases (Figure S2D, E). These results clearly demonstrate that CRC cells with different-sized nuclei are capable of being prospectively sorted into LNCs and SNCs.

### LNCs possess greater constricted migratory and metastatic potential than SNCs

**Figure 3:**
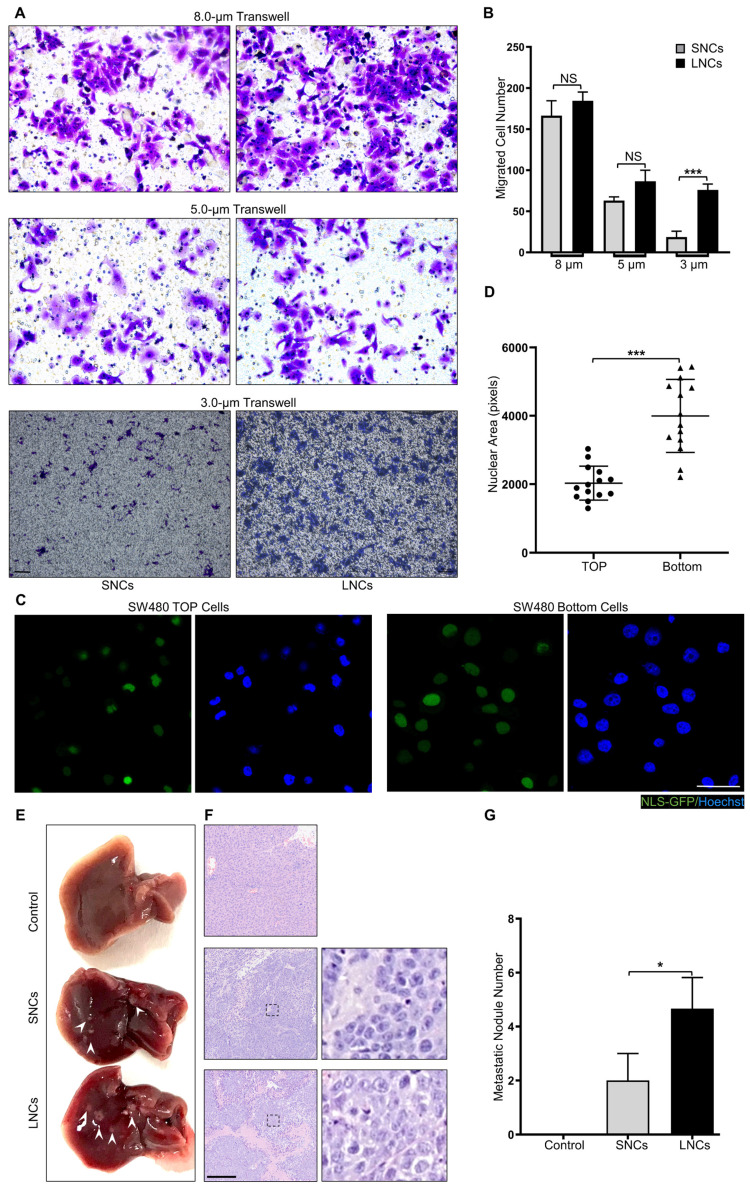
** LNCs possess greater constricted migratory and metastatic potential than SNCs.** (A) Transwell assays with 8.0-, 5.0-, and 3.0-μm pore sizes of SW480 SNCs and LNCs. Scale bar: 100 μm. (B) Quantification of migrated SW480 SNCs and LNCs. (C) Immunofluorescence analysis of NLS-GFP and nuclei (Hoechst) of SW480 cells before (TOP) and after (Bottom) the Transwell assays with a 3.0-μm pore size. Scale bar: 20 μm. (D) Quantification of the nuclear area of SW480 TOP cells and SW480 Bottom cells. (E) Liver metastases in mice of the control, SW480 SNC, and SW480 LNC groups. (F) HE staining of liver lesions in the corresponding groups. Scale bar: 100 μm. (G) Quantification of metastatic nodules. Results are presented as the mean ± SD. ^*^P < 0.05, ^***^P < 0.001.

Confined cell migration refers to the squeezing of cells through ECM pores with a subnuclear diameter 34-36. To mimic confined migration, Transwell assays with 8.0-, 5.0-, or 3.0-μm pore sizes were performed in LNCs and SNCs, where 3.0-μm pores were subnuclear size tracks 5. SW480 LNCs and SNCs migrated equivalently through 8.0- or 5.0-μm pores that minimally constrained the nucleus (Figure 3A, B). However, more LNCs than SNCs migrated through the 3.0-μm pores (Figure 3A, B). Furthermore, migrated SW480 bulk cells in the 3.0-μm pore chamber exhibited larger nuclei than those of cells in the top chamber (Figure 3C, D). Notably, LNCs and SNCs exhibited different N/C ratios. To investigate whether nuclear size or N/C ratio contribute to confined cell migration, LNCs and SNCs were further sorted into large-sized cells with large nuclei (L-L), small-sized cells with large nuclei (S-L), large-sized cells with small nuclei (L-S), and small-sized cells with small nuclei (S-S). 3.0-μm Transwell assays were performed in these cell subpopulations (Figure S3A). The N/C ratio of S-L cells was larger than that of L-L cells, whereas the N/C ratio of L-S cells was smaller than that of S-S cells (Figure S3B). However, there was no significant difference in the migrated cell number between L-L and S-L cells or between L-S and S-S cells (Figure S3C). Notably, *in vivo* assays showed that LNCs produced more liver metastases in nude mice than SNCs (Figure 3E-G). These results reveal that LNCs possess greater constricted migratory capacity and metastatic potential than SNCs.

### Constricted migratory capacity depends on the nuclear stiffness of the cells

**Figure 4:**
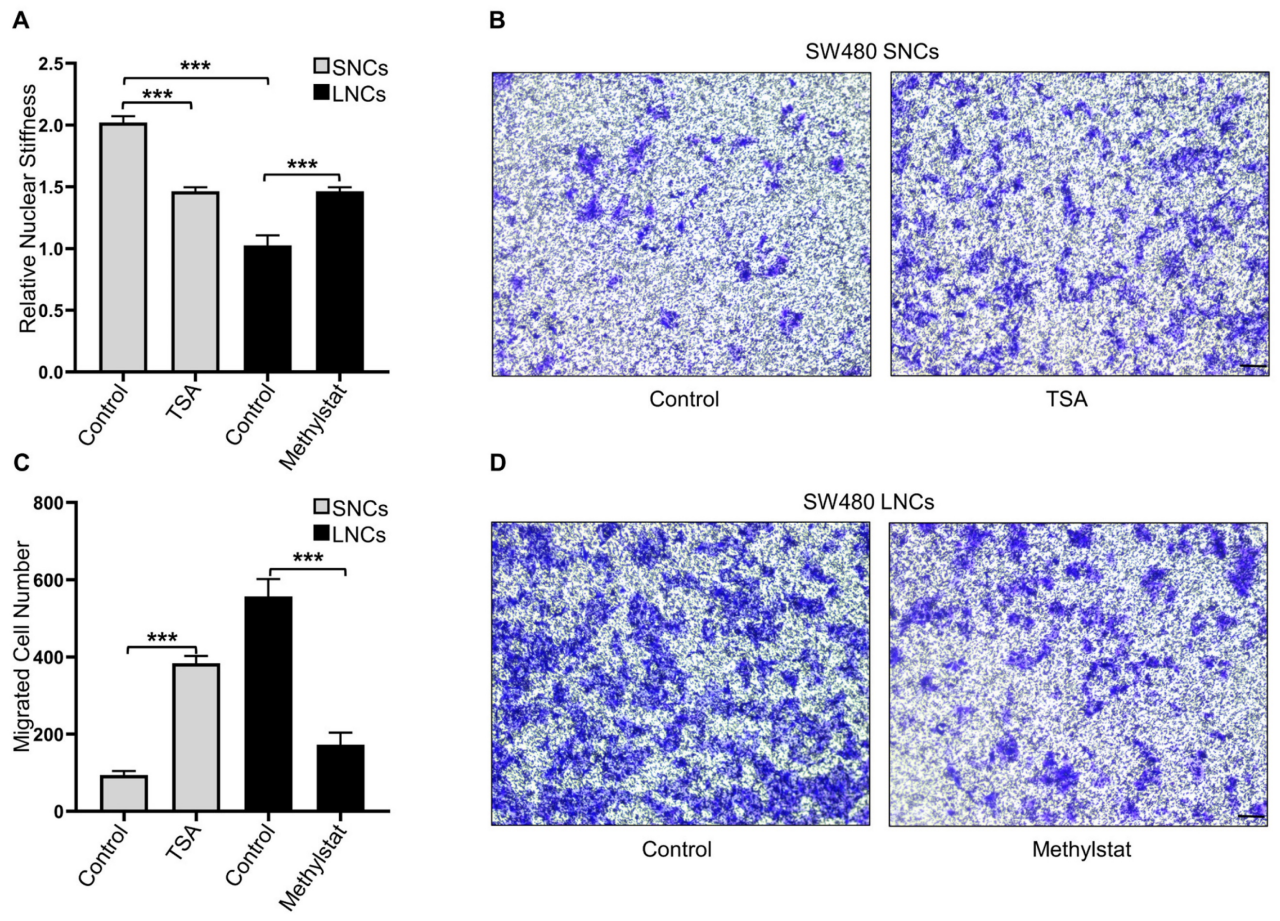
** Constricted migratory capacity depends on the nuclear stiffness of the cells.** (A) Quantification of the relative nuclear stiffness of SW480 SNCs and LNCs, treated with TSA (2 μM) or methylstat (2 μM), and DMSO as a control. (B) Transwell assays with a 3.0-μm pore size of SW480 SNCs, treated with TSA (2 μM), and DMSO as a control. Scale bar: 100 μm. (C) Quantification of migrated SW480 SNCs and LNCs, treated with TSA (2 μM) or methylstat (2 μM), and DMSO as a control. (D) Transwell assays with a 3.0-μm pore size of SW480 LNCs, treated with methylstat (2 μM), and DMSO as a control. Scale bar: 100 μm. Results are presented as the mean ± SD. ^***^P < 0.001.

Confined cell migration is associated with nuclear stiffness 17. AFM was employed to measure the nuclear stiffness of SW480 LNCs and SNCs, which indicated that the nuclei of LNCs were softer than those of SNCs (Figure 4A). To further investigate the association between nuclear stiffness and confined cell migration, SW480 SNCs were treated with TSA, a histone deacetylase inhibitor that increases euchromatin and consequently reduces nuclear stiffness 13. TSA may also elevate acetylated α-tubulin levels and thus inhibit cell motility 37. To verify the effect of TSA on CRC cells, total and acetylated α-tubulin were analyzed. The results indicated that TSA did not induce the acetylation of α-tubulin in CRC cells (Figure S4A). TSA-treated SNCs showed reduced nuclear stiffness and increased migration in the 3.0-μm Transwell assay (Figure 4A-C). The histone demethylase inhibitor methylstat increases heterochromatin and nuclear stiffness 38. Methylstat-treated LNCs displayed increased nuclear stiffness and decreased migration in the 3.0-μm Transwell assay (Figure 4A, C, and D). In conclusion, these results suggest that differences in the confined migration of SW480 LNCs and SNCs are dependent on nuclear stiffness.

### Nuclear stiffness is mediated by the ErbB4 signaling pathway

**Figure 5:**
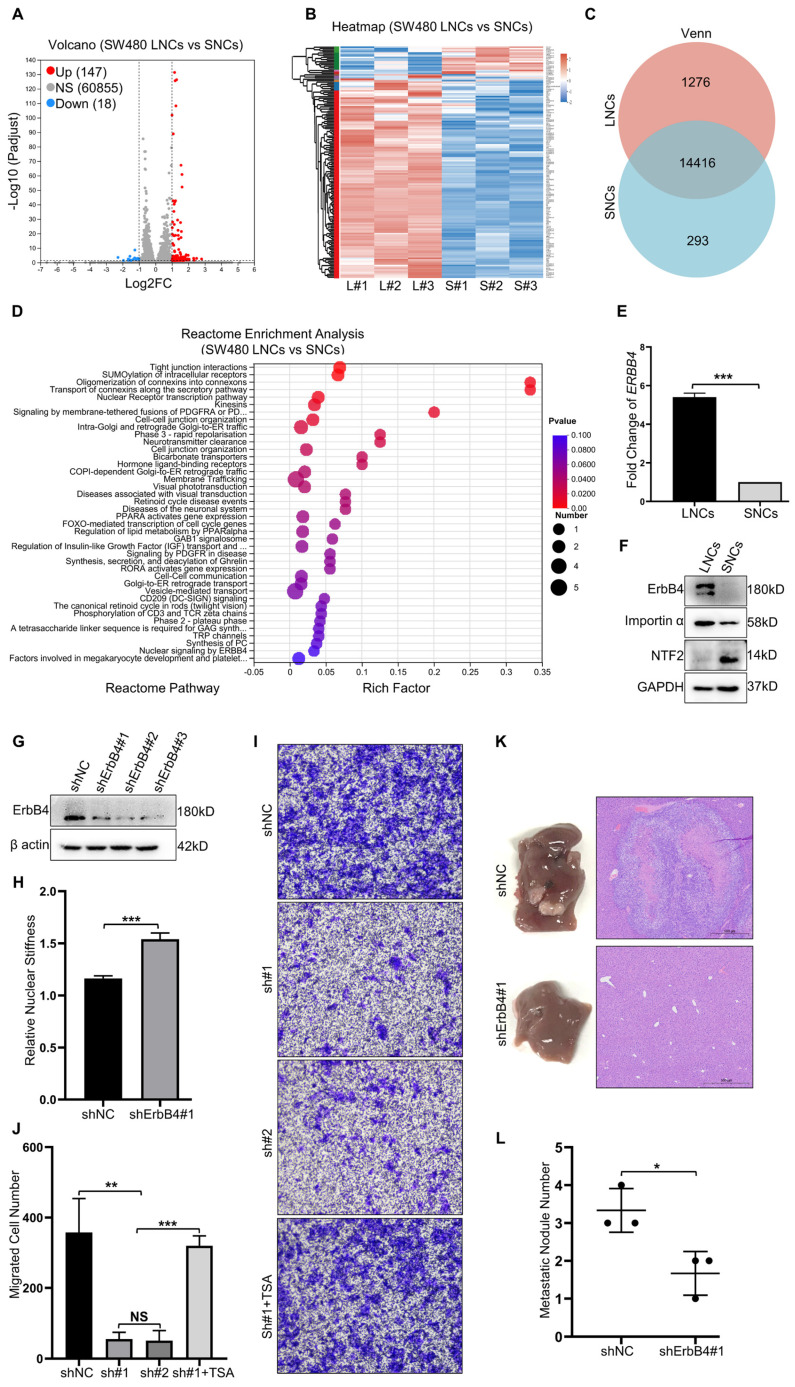
** Nuclear stiffness is mediated by the ErbB4 signaling pathway.** (A and B) Volcano plot (A) and Heat map (B) of differentially expressed genes in SW480 LNCs and SNCs. (C) Venn analysis of co-expressed genes and specifically expressed genes in SW480 LNCs and SNCs. (D) Reactome enrichment analysis of upregulated genes in SW480 LNCs. (E) Fold change in *ERBB4* levels of SW480 LNCs and SNCs determined by RT-qPCR. Fold change = 5.40. (F) Western blotting analysis of ErbB4, Importin α, and NTF2 in SW480 LNCs and SNCs. (G) Western blotting analysis to detect the knockdown of ErbB4 (shErbB4#1, #2, and #3) in SW480 cells, with shNC as a control. (H) Quantification of the relative nuclear stiffness of shNC and shErbB4#1 SW480 LNCs. (I) Transwell assays with a 3.0-μm pore size of shNC, shErbB4#1 (sh#1), and shErbB4#2 (sh#2) SW480 LNCs, treated with TSA (2 μM), and DMSO as a control. Scale bar: 100 μm. (J) Quantification of migrated shNC, shErbB4#1 (sh#1), and shErbB4#2 (sh#2) SW480 LNCs, treated with TSA (2 μM), and DMSO as a control. (K) Liver metastases in mice of the shNC and shErbB4#1 SW480 LNC groups. HE staining of the liver lesions in the corresponding groups. Scale bar: 500 μm. (L) Quantification of metastatic nodules. Results are presented as the mean ± SD. ^*^P < 0.05, ^**^P < 0.01, ^***^P < 0.001.

RNA-seq analysis of SW480 LNCs and SNCs revealed differentially expressed genes, with 147 up-regulated genes in LNCs (Figure 5A-C). Reactome enrichment analysis revealed that the upregulated genes in LNCs were involved in tight junction interactions, cell-cell junctions, membrane-tethered fusion, and nuclear signaling by *ERBB4* (Figure 5D). ErbB4 activates signaling pathways involved in tumor development 25. Once activated, the C-terminal domain of ErbB4 translocates to the nucleus and modulates nuclear signaling 26. Further investigation revealed a high level of ErbB4 at both the mRNA and protein levels in LNCs (Figure 5E, F). ShRNAs were used to knock down ErbB4 in CRC cells (Figure 5G). Notably, the nuclei of the shErbB4 SW480 LNCs were stiffer than those of the shNC group (Figure 5H). Knockdown of ErbB4 reduced the confined migration of SW480 LNCs in a 3.0-μm Transwell assay, which was rescued by TSA treatment (Figure 5I, J). Consistent with Transwell assays, the shNC SW480 LNCs exhibited more liver metastases than the shErbB4 SW480 LNCs (Figure 5K, L). These results indicate that ErbB4, which is highly expressed in LNCs, contributes to decreased nuclear stiffness and thus increases metastasis in CRC.

### Akt1 directly interacts with and phosphorylates lamin A/C at Ser22

**Figure 6:**
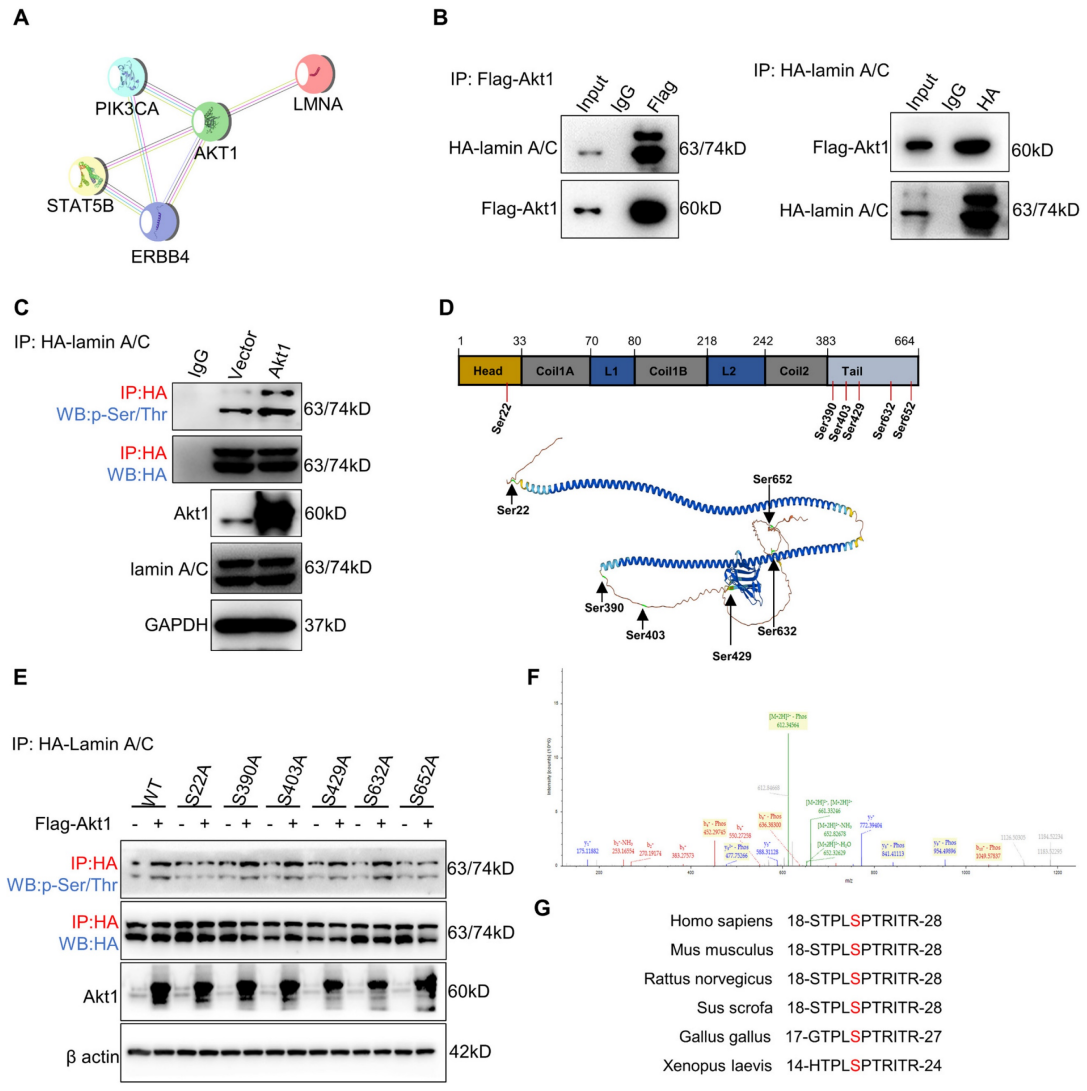
** Akt1 directly interacts with and phosphorylates lamin A/C at Ser22.** (A) ErbB4 and lamin A/C interaction networks in the STRING database. (B) CoIP analysis showing the binding of Flag-Akt1 and HA-lamin A/C in the HEK293T cell line. (C) QIP to identify the lamin A/C and phosphorylated lamin A/C (p-Ser/Thr) changes with overexpression of Akt1 in the HEK293T cell line, using vector as a control. (D) MS analysis of the phosphorylated serines of HA-lamin A/C in the HEK293T cell line. (E) QIP assays were performed to detect the phosphorylation (p-Ser/Thr) of wild-type and S-A mutant HA-lamin A/C in the HEK293T cell line, with or without Akt1 overexpression. (F) Secondary MS results of phosphorylation of lamin A/C at Ser22. (G) Amino acid at Ser22 of lamin A/C in different species.

Nuclear stiffness is modulated by the nuclear fibrillar protein lamin A/C 39. Lamin A/C depletion or phosphorylation decreases nuclear stiffness and enhances confined migration 18, 40. To investigate the potential impact of ErbB4 on lamin A/C, the STRING database analysis was performed. The analysis revealed that Akt1 may regulate the interaction network between ErbB4 and lamin A/C (Figure 6A). Flag-Akt1 and HA-lamin A/C plasmids were constructed and co-transfected into the HEK293T cell line. CoIP assays indicated that Akt1 directly interacted with lamin A/C (Figure 6B). Akt1 is a serine/threonine-protein kinase that phosphorylates the substrate proteins. A quantitative IP assay was performed in the HEK293T cell line co-transfected with Flag-Akt1 and HA-lamin A/C. The overexpression of Akt1 was associated with the hyperphosphorylation of lamin A/C, but there was little or no alteration in the level of total lamin A/C (Figure 6C).

To identify the lamin A/C phosphorylation site(s), lamin A/C was enriched by IP for MS analysis, and six serine (Ser) residues were identified (Figure 6D). To figure out the target site of the Akt1-dependent lamin A/C phosphorylation residue, Ser to alanine (A) phosphorylation-defective mutant plasmids at the six residues were constructed, respectively. Quantitative IP was used to detect the phosphorylation status of lamin A/C by overexpressing Akt1. The results demonstrated that the S22A mutation attenuated the phosphorylation of lamin A/C (Figure 6E). Notably, a secondary MS result confirmed the phosphorylation of lamin A/C at Ser22 (Figure 6F), which was found to be conservative in most species (Figure 6G). In conclusion, Akt1 directly interacts with and phosphorylates lamin A/C at the Ser22 residue.

### ErbB4-Akt1-lamin A/C signaling mediates the confined migration of CRC cells

**Figure 7:**
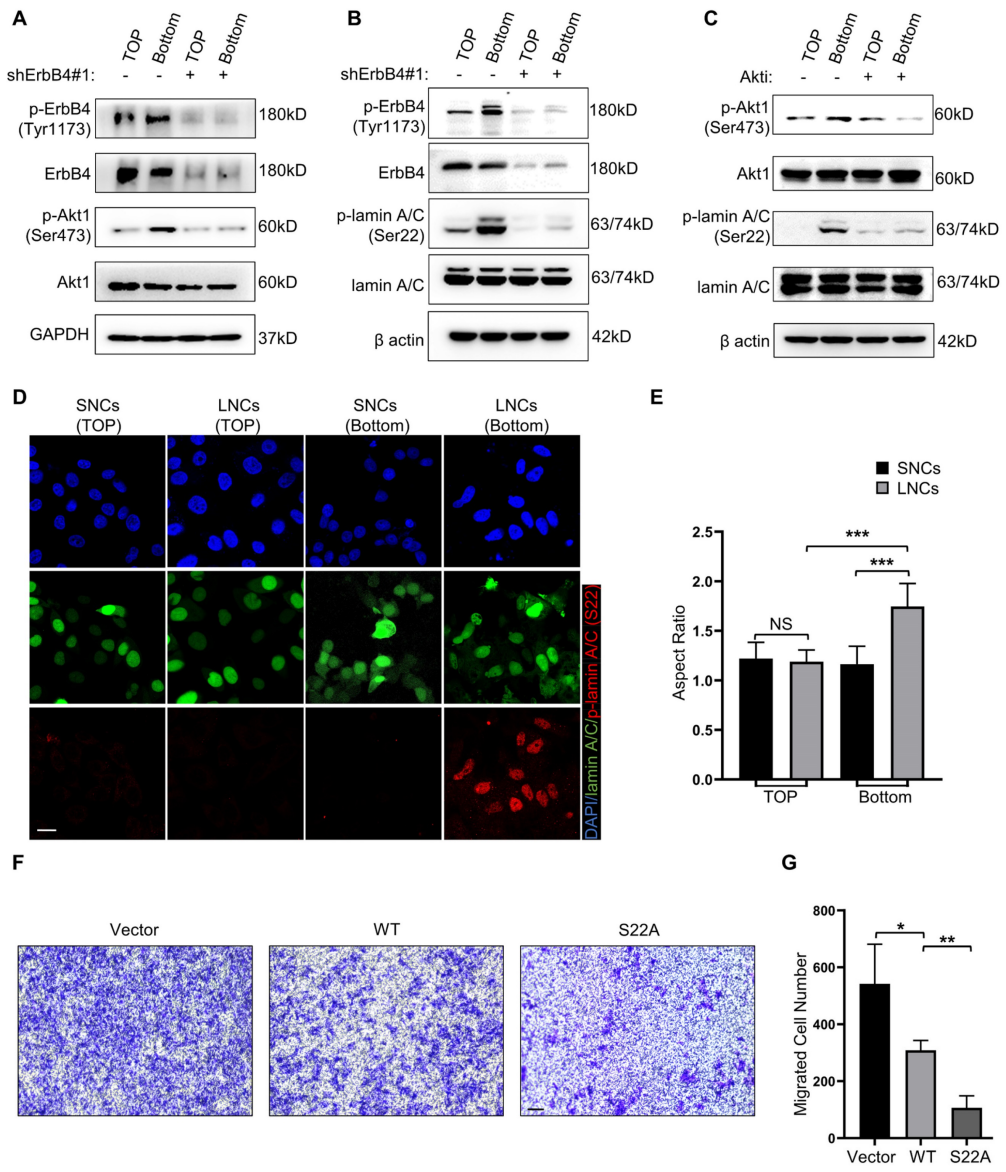
** ErbB4-Akt1-lamin A/C signaling mediates the confined migration of CRC cells.** (A) Western blotting analysis of ErbB4, p-ErbB4 (Tyr1173), Akt1, and p-Akt1 (Ser473) in SW480 LNCs treated with or without shErbB4#1, before (TOP) and after (Bottom) the Transwell assays. (B) Western blotting analysis of ErbB4, p-ErbB4 (Tyr1173), lamin A/C, and p-lamin A/C (Ser22) in SW480 LNCs treated with or without shErbB4#1, before (TOP) and after (Bottom) the Transwell assays. (C) Western blotting analysis of Akt1, p-Akt1 (Ser473), lamin A/C, and p-lamin A/C (Ser22) in SW480 LNCs treated with or without Akti (0.5 μM), before (TOP) and after (Bottom) the Transwell assays. (D) Immunofluorescence analysis of lamin A/C, p-lamin A/C (Ser22), and nuclei (DAPI) of SW480 SNCs and LNCs, before (TOP) and after (Bottom) the Transwell assays. Scale bar: 20 μm. (E) Quantification of the nuclear aspect ratio of SW480 SNCs and LNCs, before (TOP) and after (Bottom) the Transwell assays. (F) Transwell assays with a 3.0-μm pore size were performed in shLamin A/C SW480 LNCs transfected with either vector, wild-type (WT), or S22A mutant lamin A/C. Scale bar: 100 μm. (G) Quantification of the migrated cell number. Results are presented as the mean ± SD. ^*^P < 0.05, ^**^P < 0.01, ^***^P < 0.001.

To explore whether ErbB4-Akt1-lamin A/C mediates the confined migration of CRC, Transwell assays with a 3.0-μm pore size were performed in SW480 LNCs. The results indicated that migrated LNCs expressed high levels of phosphorylated ErbB4 and Akt1 (Figure 7A). In addition, the phosphorylation of Akt1 was markedly reduced upon knockdown of ErbB4 (Figure 7A). Furthermore, lamin A/C was phosphorylated at Ser22 in migrated LNCs, which was reversed by the knockdown of ErbB4 (Figure 7B). The Akt kinase inhibitor (Akti) was used to inhibit Akt1, resulting in a decrease of lamin A/C phosphorylation at Ser22 in migrated LNCs (Figure 7C). Besides the Ser22 site, some other residues of lamin A/C, such as Ser390 and Ser404, were reported to be phosphorylated by Akt 41, 42. Western blotting analysis indicated that Ser22 was the predominant phosphorylation site in migrated LNCs (Figure S4B). Consistently, the IF assay indicated the phosphorylation of lamin A/C at Ser22 in migrated LNCs, and migrated LNCs expressed more phosphorylated lamin A/C than SNCs (Figure 7D). Notably, the nuclei of both SNCs and LNCs were rounded before migration, but the nuclei of migrated LNCs were deformed and exhibited an elevated aspect ratio, while the nuclei of migrated SNCs remained typically rounded, indicating that the nuclei of LNCs were indeed softer (Figure 7D, E). To further identify the function of phosphorylation at Ser22 of lamin A/C, a lamin A/C knockdown cell line was constructed using shRNAs and rescued with either wild-type lamin A/C or S22A mutant lamin A/C. The Transwell assay results revealed that S22A mutant lamin A/C partially attenuated the confined migration of CRC cells (Figure 7F, G). Taken together, these results suggest that lamin A/C is phosphorylated at Ser22 in CRC cells via the ErbB4-Akt1 pathway, and the confined migration of CRC cells is inhibited by the phosphorylation-defective mutant S22A of lamin A/C.

### ERBB4 is upregulated in CRC and positively associated with CRC stage and poor prognosis

**Figure 8:**
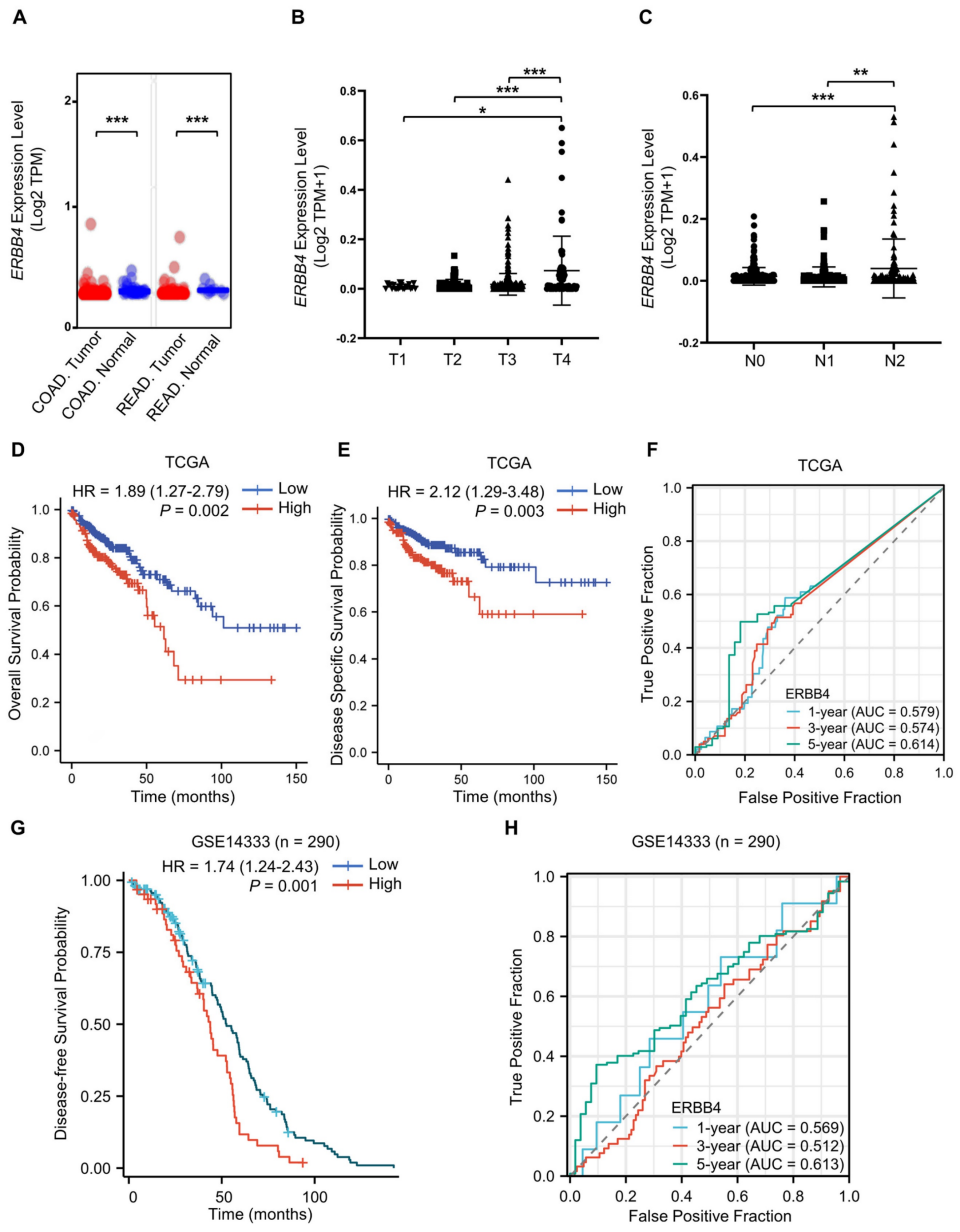
** ERBB4 is upregulated in CRC and positively associated with CRC stage and poor prognosis.** (A) The expression status of* ERBB4* between normal tissues and tumor tissues of COAD and READ in the TCGA database. (B) Based on the TCGA database, the expression level of *ERBB4* was analyzed by the TNM stages (T1, T2, T3, and T4) of CRC. (C) Based on the TCGA database, the expression level of *ERBB4* was analyzed by the TNM stages (N0, N1, and N2) of CRC. (D and E) Overall survival (D) and disease-specific survival (E) analyses of CRC in TCGA by *ERBB4* expression. (F) Based on the TCGA database, ROC curves and associated AUC values in CRC with *t* = 1, 3, and 5 years. (G) Based on the GSE14333 dataset, disease-free survival analysis of CRC by *ERBB4* expression. (H) Based on the GSE14333 dataset, ROC curves and associated AUC values in CRC with *t* = 1, 3, and 5 years. Results are presented as the mean ± SD. ^*^P < 0.05,^ **^P < 0.01, ^***^P < 0.001.

To clarify the role of ErbB4 in tumorigenesis and progression, the expression status of *ERBB4* in the CRC of the TCGA database was analyzed using the UALCAN tool 43. The expression level of *ERBB4* in the tumor tissues of colon adenocarcinoma (COAD) and rectum adenocarcinoma (READ) was higher than the corresponding normal tissues (Figure 8A). The level of *ERBB4* in CRC at the different TNM stages was analyzed. *ERBB4* was upregulated in the T4 and N2 stages of CRC (Figure 8B, C). Analyses of the survival data in the TCGA database and the GSE14333 dataset revealed poor overall survival, disease-specific survival, or disease-free survival in the high *ERBB4* CRC groups (Figure 8D, E, and G). Time-dependent ROC curves demonstrated that *ERBB4* expression possessed a certain degree of predictive performance for the survival of CRC patients (Figure 8F, H).

## Discussion

In the present study, we confirmed that nuclear size was positively associated with the stage of CRC. Moreover, CRC LNCs possessed a higher degree of constricted migratory and metastatic capacity than SNCs, due to the soft nuclei in LNCs. Furthermore, we revealed that ErbB4 was highly expressed in LNCs and phosphorylated lamin A/C at Ser22 through the ErbB4-Akt1 pathway, which was responsible for nuclear softening and enhanced confined cell migration.

Tumors consist of a heterogeneous collection of cells that selectively facilitate metastasis 44. Increased metastasis correlates with smaller cellular size or larger nuclear size in the colon, breast, and several other cancer types 8, 12, 45-47. Therefore, cell size, or nucleus size, is used to assess tumor malignancy. Considering small cells and large nuclei together, it is possible that the disrupted N/C ratio is a reliable biomarker for aggressive cancers 33, 48. The N/C ratio scales the nuclear size through NE mechanics and regulates nuclear mobility through the interaction between the cytoskeleton and NE proteins. In our study, we identified nuclear size as an indicator that had a more direct and impactful influence on metastasis, particularly on confined cell migration, instead of cell size or N/C ratio. These findings provided a theoretical basis for the use of nuclear size to assess CRC metastasis.

Lamin A/C forms the nuclear lamina on the NE and influences metastasis via modulating nuclear stiffness in several ways. First, increasing nuclear size without a corresponding increase in the total amount of lamin A/C results in a thinner and more malleable NE 17, 33. Deletion or inhibition of lamin A/C leads to increased nuclear deformability and cell migration in confined environments 49-52. Second, lamin A/C phosphorylation plays a vital role in regulating lamina stability and, subsequently, nuclear stiffness 53, 54. The Ser22 site of lamin A/C is phosphorylated by MAPK and PKC kinases, which are targets of nuclear size-regulating compounds 55, 56. Finally, lamin A/C is mechano-responsive to ECM elasticity. Mechanical force acting on the nucleus leads to conformational changes and increased phosphorylation of lamin A/C 17, 21, 57. Our findings revealed that the soft nuclei of LNCs were dependent on laminA/C phosphorylation. Phosphorylation of lamin A/C at Ser22 by Akt1 kinase, without a significant reduction of total lamin A/C, promoted confined migration of CRC cells.

The mechanical force from the ECM must be transmitted to the nucleus and, hence, participate in the phosphorylation of lamin A/C 49, 58. Mechanical signals are transduced to receptor tyrosine kinases in the ErbB family, contributing to the activities of the ErbB family 27. Once activated, ErbB4 is capable of activating multiple kinases, including Akt1, MAPK, and PI3K 59-61. Furthermore, the soluble intracellular domain of ErbB4 translocates to the nucleus and activates gene transcription 26, 62. Along these lines, our study revealed that ErbB4 was activated in CRC LNCs during confined cell migration and phosphorylated lamin A/C at Ser22 through the ErbB4-Akt1 pathway.

In summary, the present study demonstrated that CRC LNCs possessed greater constricted migratory and metastatic capacity than SNCs due to nuclear stiffness, which was dependent on the ErbB4-Akt1-lamin A/C pathway. Certainly, it is difficult to adequately reproduce the physical constriction of nuclei and witness the dynamic process of nuclear deformations. However, the results of the present study truly provide new perspectives for the regulation of the nucleus and metastasis by physical factors, which can be used as targets in targeting cancer metastasis.

## Supplementary Material

Supplementary figures and tables.

## Abbreviations

AFM: atomic force microscopy
Akti: Akt kinase inhibitor
BCS: body condition score
BSA: bovine serum albumin
COAD: colon adenocarcinoma
CoIP: co-immunoprecipitation
CRC: colorectal cancer
ECM: extracellular matrix
FACS: fluorescence-activated cell sorting
FSC: forward scatter
GFP: green fluorescent protein
HE: hematoxylin and eosin
IF: immunofluorescence
LNCs: large-nucleated cells
MS: mass spectrometry
N/C ratio: nucleus-to-cell volume ratio
NE: nuclear envelope
NLS: nuclear localization sequence
PI: propidium iodide
qIP: quantitative immunoprecipitation
READ: rectum adenocarcinoma
RNA-seq: RNA-sequencing
RT-qPCR: reverse transcription-quantitative polymerase chain reaction
SNCs: small-nucleated cells
Taxol: Paclitaxel
TNM: tumor-node-metastasis
TSA: trichostatin A
XhCRC: colorectal cancer xenograft
