# Supplementary material for: Nuclear Softness Promotes the Metastatic Potential of Large-Nucleated Colorectal Cancer Cells via the ErbB4-Akt1-Lamin A/C Signaling Pathway
Source: Int J Biol Sci. 2024 Apr 29;20(7):2748–62. doi: 10.7150/ijbs.89481 (PMC11077370; doi:10.7150/ijbs.89481)
Supplement: Supplementary file 1 — Supplementary figures and tables. [file ijbsv20p2748s1.pdf]

Supplementary Tables

Table S1. Clinical history of human subjects.

Patient No.	Gender	Age	TNM	Tumor Stage
CRC1	M	63	T2N0M0	I
CRC2	M	48	T1N0M0	I
CRC3	M	62	T3N0M0	II
CRC4	F	57	T3N1aM0	III
CRC5	F	65	T2N0M0	I
CRC7	M	47	T3N0M0	II
CRC8	M	64	T3N0M0	II
CRC9	F	67	T3N1M0	III
CRC10	F	67	T3N1cM0	III
CRC14	M	61	T4N0M1	IV
CRC18	M	67	T1N0M1	IV
CRC22	M	48	T4N1M1	IV
CRC6	M	63	T4bN1M1	IV
CRC24	M	64	T3N1cM1	IV
CRC26	F	64	T4N2M1	IV
CRC28	M	64	T3N0M1	IV
CRC29	F	58	T3N2M1	IV
CRC35	M	70	T4N0M1	IV
CRC39	F	64	T4N2M1	IV
CRC42	M	67	T3N0M1	IV
XhCRC	F	47	T4N2M0	III

Table S2. Antibodies and Reagents used in experimental procedures.

Reagent or Resource	Company	Cat.#	Dilution
**Antibodies**			
lamin A/C	Cell Signaling Technology	4777	1:1 000
phospho-lamin A/C (Ser22)	Cell Signaling Technology	13448	1:1 000
phospho-lamin A/C (Ser390)	Thermo Fisher Scientific	PA5-114596	1:1 000
phospho-lamin A/C (Ser404)	Sigma-Aldrich	ABT1387	1:500
ErBb4	Proteintech	19943-1-AP	1:1 000
phospho-ErBb4 (Tyr1173)	Cell Signaling Technology	4757	1:1 000
Akt1	Cell Signaling Technology	2938	1:1 000
phospho-Akt1 (Ser473)	Cell Signaling Technology	4060	1:1 000
phospho-Ser/Thr	ABclonal	AP0893	1:500
α-tubulin	ABclonal	AC012	1:1 000
Acetyl-α-tubulin (Lys40)	Cell Signaling Technology	5335	1:1 000
NTF2	ABclonal	A7057	1:1 000
Importin α	ABclonal	A5012	1:1 000
GAPDH	Abcam	ab9484	1:1 000
β actin	Abcam	ab6276	1:1 000
Goat Anti-Mouse IgG, DyLight 488	Abbkine	A23210	1:100

Goat Anti-Rabbit IgG, Cy3	Abbkine	A22220	1:100
Goat Anti-Mouse IgG, HRP	Abbkine	A21010	1:5 000
Goat Anti-Rabbit IgG, HRP	Abbkine	A21020	1:5 000
**Reagents**			
Trichostatin A (TSA)	MedChemExpress	HY-15144	2 μM
methylstat	MedChemExpress	HY-15221	2 μM
Akt kinase inhibitor (Akti)	MedChemExpress	HY-10249A	0.5 μM
paclitaxel (Taxol)	MedChemExpress	HY-B0015	20 nM
Rnase A	Thermo Fisher Scientific	EN0531	10 mg/mL
Propidium Iodide (PI)	Thermo Fisher Scientific	P1304MP	50 μg/mL
Hoechst 33342	MedChemExpress	HY-15559	10 μg/mL

Table S3. Sequences of the primers used for PCR and RT-qPCR.

	Genes	Oligo sequences (forward) (5' to 3')	Oligo sequences (reverse) (5' to 3')
PCR	*LMNA-HA*	GGCTAGCGTTTAAACGGGCCCATGG<br>AGACCCCGTCCCAGC	GGGTAACTTAAGCTTGGTACCGACATGATG<br>CTGCAGTTCTGGG
RT-qPCR	*ERBB4*	GTCCAGCCCAGCGATTCTC	AGAGCCACTAACACGTAGCCT
RT-qPCR	*GAPDH*	GGAGCGAGATCCCTCCAAAAT	GGCTGTTGTCATACTTCTCATGG

Supplementary figures and figure legends

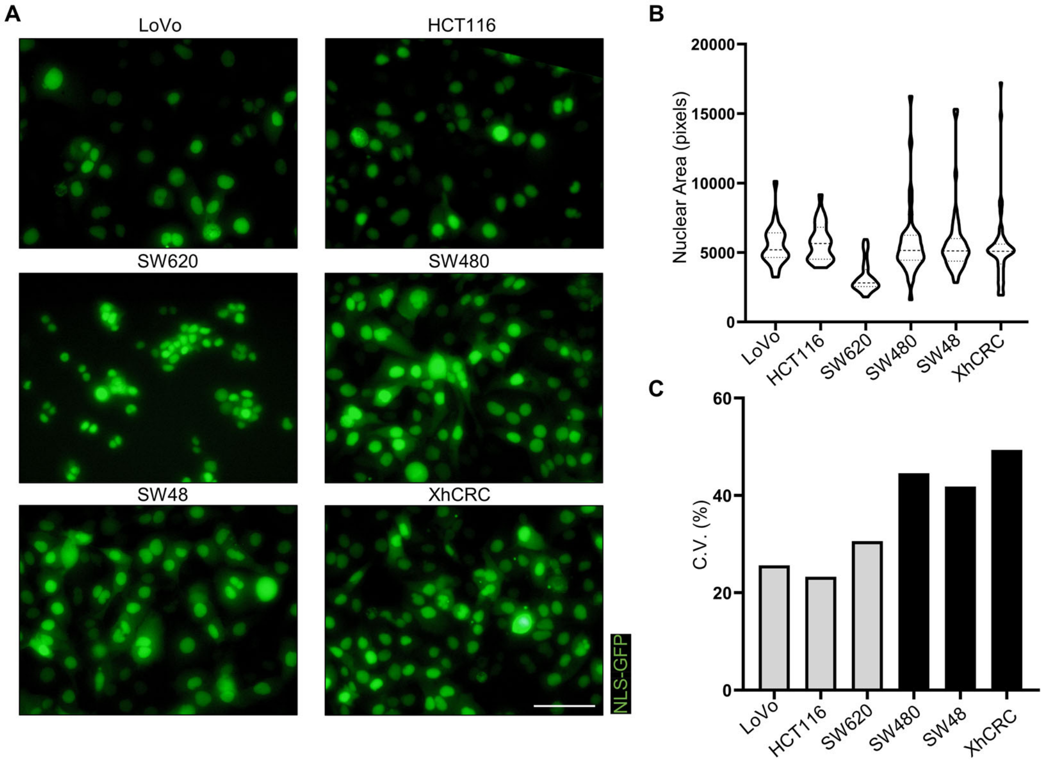

Figure S1. Heterogeneity of nuclear size in the CRC cell lines. (A) Immunofluorescence analysis of NLS-GFP of the CRC cell lines. Scale bar: 20 μm. (B and C) Violin plot analysis (B) and coefficient of variation (C.V.) analysis (C) of the nuclear area of the CRC cell lines.

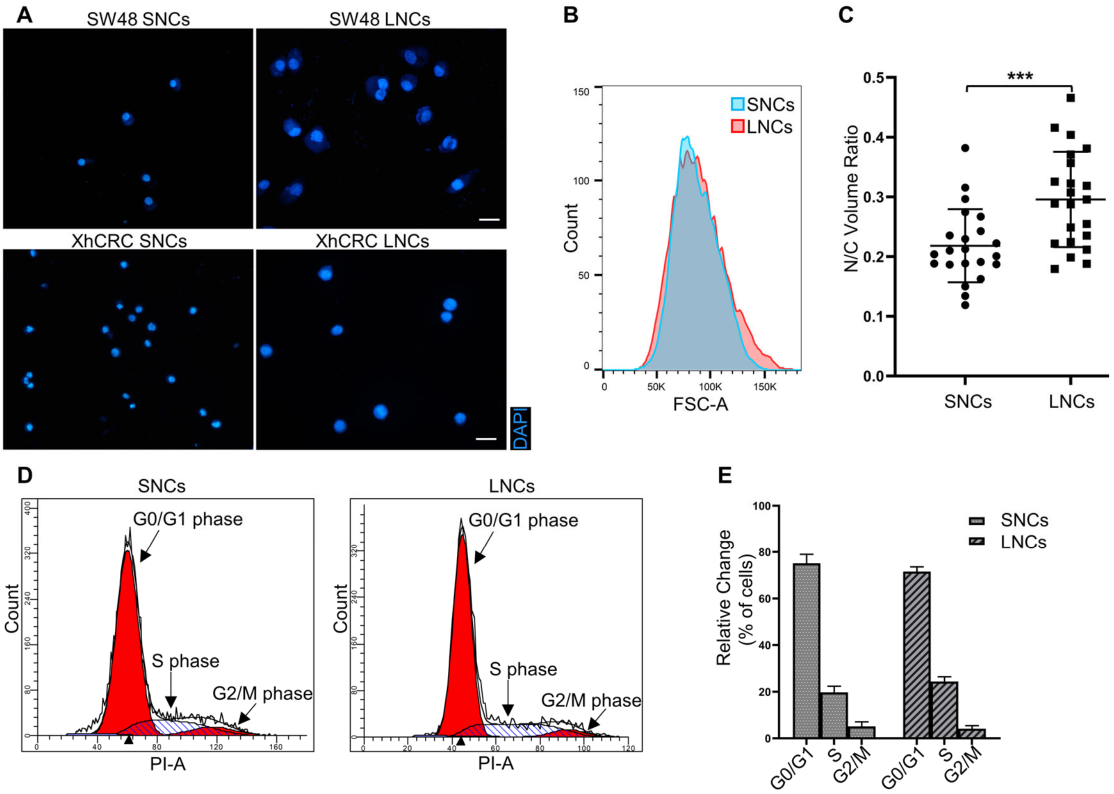

Figure S2. LNCs possess larger nuclei and higher nucleus-to-cell volume (N/C) ratios than SNCs. (A) Immunofluorescence analysis of nuclei (DAPI) of SNCs and LNCs in the SW48 and XhCRC cell lines. Scale bar: 20 μm. (B) Flow cytometry analysis based on FSC-A was performed to examine the cell size of SW480 SNCs and LNCs. (C) Quantification of the N/C ratio of SW480 SNCs and LNCs. (D) Flow cytometry analysis based on PI-A was performed to detect the cell cycle of SW480 SNCs and LNCs. (E) The percentage of SW480 SNCs and LNCs in different cell cycle phases. Results are presented as the mean ± SD. $^{***}P$ <0.001.

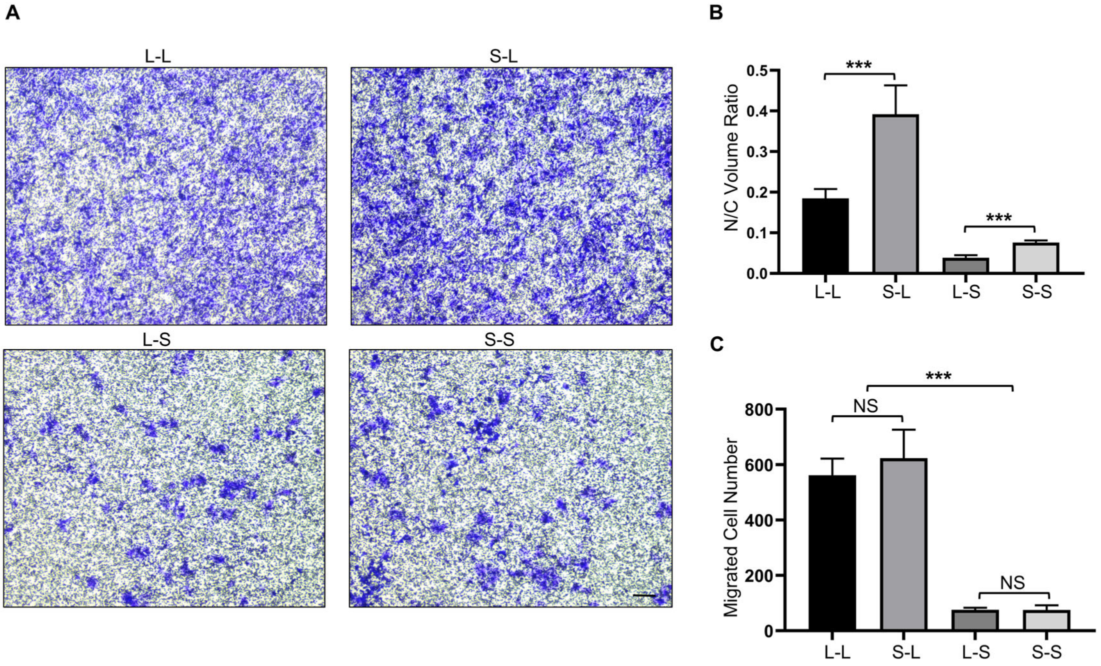

Figure S3. LNCs possess greater constricted migratory capacity than SNCs, regardless of their cell size or N/C ratio. (A) Transwell assays with a 3.0-μm pore size of SW480 large-sized cells with large nuclei (L-L), small-sized cells with large nuclei (S-L), large-sized cells with small nuclei (L-S), and small-sized cells with small nuclei (S-S), respectively. Scale bar: 100 μm. (B) Quantification of the N/C ratio of SW480 L-L cells, S-L cells, L-S cells, and S-S cells. (C) Quantification of migrated cell number. Results are presented as the mean ± SD. $^{***}P < 0.001$.

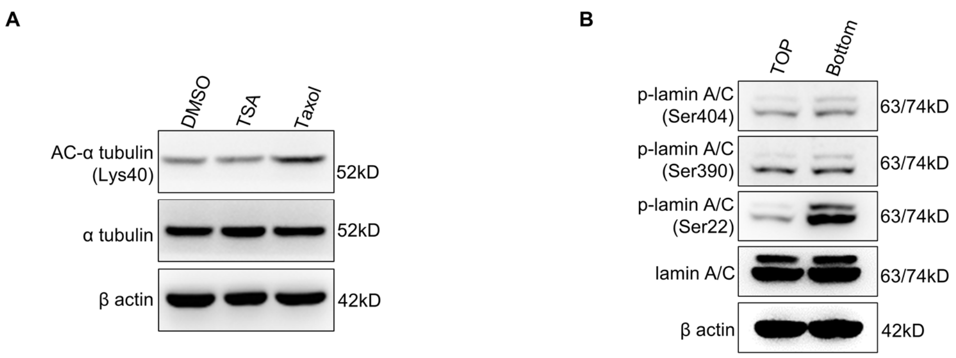

Figure S4. Western blotting of the acetylation of α tubulin and the phosphorylation of lamin A/C. (A) Western blotting analysis to detect α tubulin and acetylated-α tubulin (AC-α tubulin, Lys40) in SW480 cells treated with TSA (2 μM), DMSO or Taxol (20 nM) as the control. (B) Western blotting analysis to detect lamin A/C, p-lamin A/C (Ser22), p-lamin A/C (Ser390), and p-lamin A/C (Ser404) in SW480 LNCs before (TOP) and after (Bottom) the Transwell assays.
